# Impact and Enablers of Pharmacogenetic-Informed Treatment Decisions—A Longitudinal Mixed-Methods Study Exploring the Patient Perspective

**DOI:** 10.3390/pharmacy13010014

**Published:** 2025-01-31

**Authors:** Anna Bollinger, Melissa Semedo Fortes, Henriette E. Meyer zu Schwabedissen, Kurt E. Hersberger, Céline K. Stäuble, Samuel S. Allemann

**Affiliations:** 1 Pharmaceutical Care Research Group, Department of Pharmaceutical Sciences, University of Basel, 4056 Basel, Switzerland; m.semedofortes@stud.unibas.ch (M.S.F.); kurt.hersberger@unibas.ch (K.E.H.); celine.staeuble@unibas.ch (C.K.S.); s.allemann@unibas.ch (S.S.A.); 2 Biopharmacy, Department of Pharmaceutical Sciences, University of Basel, 4056 Basel, Switzerland; h.meyerzuschwabedissen@unibas.ch; 3 Institute of Hospital Pharmacy, Stadtspital Zurich, 8063 Zurich, Switzerland

**Keywords:** PGx, medication changes, patient perspective, implementation

## Abstract

Pharmacogenetic (PGx) testing is a promising approach for optimizing drug therapies. However, there is limited knowledge regarding its real-world utilization and long-term impact in clinical practice. This study assessed how often PGx information informs treatment decisions and evaluated patients’ perspectives on its use and non-use, identifying enablers for PGx implementation. A mixed-methods study was conducted with 24 patients with a median of 1 year after PGx testing. Medication and health-related data were collected at enrollment and at the follow-up 1 year later using a semi-structured questionnaire. At the follow-up, 62 medication changes were identified in 18 patients. A median of four medication changes per patient were initiated mainly by medical specialists (58%). PGx information was considered for 15 patients in 39 medication changes (63%). Patient-reported factors contributing to the non-use of PGx information included a lack of knowledge and interest among healthcare professionals (HCPs), structural and administrative barriers, and an over-reliance on patient advocacy. Potential enablers should address targeted PGx education, interprofessional collaboration, awareness among policymakers, and concise recommendations focused on PGx-actionable drugs from testing providers. By implementing these interdependent enablers, PGx can evolve into a long-term, clinically integrated cornerstone of individualized pharmacotherapy.

## 1. Introduction

Adverse drug reactions (ADR) and therapy failure (TF) are common drug-related problems (DRPs) [1]. Such interindividual drug responses affect a substantial part of the population. In Europe, around 8.6 million unplanned hospital admissions are caused by ADR each year, half of which are considered preventable [2].

One factor contributing to interindividual drug responses is variations in genes, which influence the pharmacokinetics (PK) and pharmacodynamics (PD) of a drug. Such genetic variations are frequent. According to a large study based on data from the UK Biobank, up to 100% of the population carry genetic variations that could lead to an interindividual response to at least one drug [3]. Meanwhile, exposure to drugs potentially affected by pharmacogenetics (PGx) is also common [4]. Data from a representative sample of the Swiss population indicate that 78.8% were exposed to at least one PGx-associated drug over the period from 2016 to 2020 [5].

Over the last three decades, personalized medicine has experienced an extensive evolution considering the increase in knowledge about genetic variations affecting drug response. In this context, PGx testing has been established to incorporate an individual’s genetic information into drug therapy decisions. Today, various providers offer PGx tests that enable personalized therapy recommendations. As a result, the “one size fits all” approach is slowly becoming obsolete, and the importance of tailored, individualized pharmacotherapy is constantly increasing. While 313 studies indexed under the Medical Subject Headings (MeSH) term “pharmacogenomics” were published in 2003, this number increased to 1111 publications in 2023 (https://pubmed.ncbi.nlm.nih.gov/, accessed on 30 January 2025).

Several studies focus on the implementation of PGx in clinical practice and assess the potential benefit of pharmacotherapies in diverse patient populations [6].

In theory, PGx testing seems to be a promising approach for optimizing drug therapies [7].

However, outside controlled study settings, it is considerably less used and accepted, and faces challenges during application [8,9,10,11]. It is also questioned whether the real-world implementation of PGx, in terms of cost and effort, is justified by the resulting improvement in therapy outcomes [6,12,13,14]. Also, there is limited knowledge about the long-term utilization and impact of PGx testing information in routine clinical practice.

In this study, we aimed to assess, in patients with access to PGx test information, how often this information is used over time to inform treatment decisions and to evaluate the factors that influence its use or non-use. Our overall goal was to provide insights into the use and non-use of PGx information and its context from the patient’s perspective and to contribute to the expert field by identifying enablers in the overall implementation process of PGx testing in clinical practice.

## 2. Materials and Methods

### 2.1. Study Design

We performed a convergent parallel mixed-method study using semi-structured interviews. This design was chosen because it enables a comprehensive exploration of the research question. The quantitative data provide measurable and numerical insights, while the qualitative data offer a deeper understanding of patients’ reported reasons and perspectives regarding the use and non-use of PGx information for treatment decisions. This approach facilitates data triangulation and enhances the validity of the findings.

**Figure 1:**
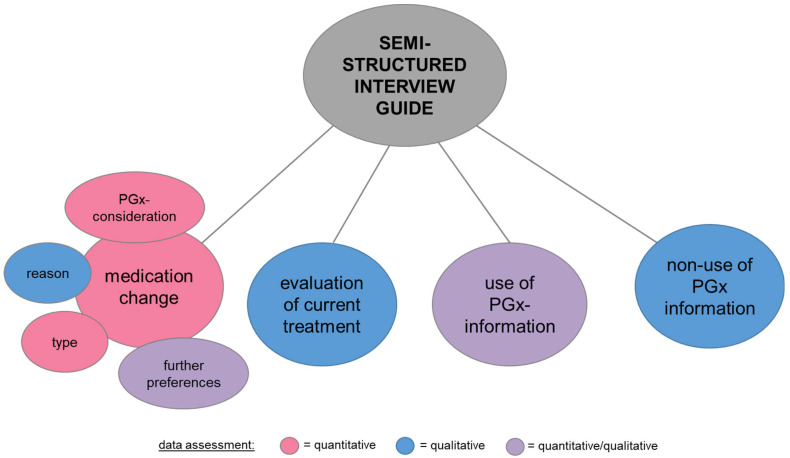
Thematic construction of the semi-structured interview guide. Abbreviation: PGx, pharmacogenetics.

We developed semi-structured interview guides with six closed and five open questions (Supplementary Materials File S1). The semi-structured interview guide addressed the following four key themes (Figure 1):

- Medication changes: For the assessment of the types of medication changes, previously defined categories [15,16] were used, as follows: 1. starting the use of a new drug; 2. discontinuing the use of a drug; 3. starting/stopping a new drug; 4. increasing a dosage; 5. decreasing a dosage; and 6. other. Additionally, the consideration of PGx information for each medication change, as well as the initiator, was structurally evaluated. The patient-reported reason for each medication change was qualitatively analyzed. Furthermore, it was quantitatively assessed whether patients had preferences for further medication changes. The underlying reasons were qualitatively explored.
- Evaluation of current treatment: the evaluation of the current treatment was qualitatively assessed, differentiating between reasons for treatment satisfaction and dissatisfaction.
- Use of PGx information: The contextual use of PGx information was qualitatively explored, focusing on the scenarios, settings, and individuals involved within the last 12 months. In addition, it was quantitatively determined whether PGx information was used in clinical practice beyond medication changes.
- Non-use of PGx information: patient-reported reasons and factors contributing to the non-use of the PGx information were qualitatively assessed.

The interview guide is available from the authors upon request.

### 2.2. Study Population

Study participants were recruited in 2023 as part of the national single-center study “Pharmacogenetic Panel Testing over time: a prospective longitudinal observational study”, which was approved by the local ethics committee in Switzerland (Ethikkomission Nordwest-und Zentralschweiz, EKNZ 2023-00157) on 21 March 2023. Patients who received PGx testing in primary or secondary healthcare institutions, either in a study setting or routine practice, were invited to participate. A requirement for participation was the patients’ access to PGx panel test information or results from at least three different PGx single gene tests. There was no time restriction on when the PGx test was conducted. All participants provided written consent for the analysis and publication of their responses.

### 2.3. Data Collection

After patient enrollment, medication- and health-related data were collected via initial telephone interviews conducted between the patient and a trained professional from the study center in 2023. This enrollment procedure ensured a consistent baseline, as the recruited patients had undergone PGx testing at different timepoints over several years before enrollment.

Follow-ups were performed as semi-structured telephone interviews one year after enrollment. Patients also had the possibility of answering the questions in written form. In this case, patients sent the completed semi-structured questionnaire, along with an updated current medication plan, to the study center using a postage-paid envelope.

Data were collected by A.B. (PhD student in Pharmaceutical Sciences) and documented using a Microsoft Office Professional Plus, Word, version 16.0 (2016) template of the interview guide. Subsequently, M.S.F. (a master’s student in Pharmacy) transferred and securely stored the data in REDCap™, a web application for building and managing study data.

### 2.4. Data Analysis

Quantitative and qualitative data were analyzed simultaneously. The Swiss trade names of the drugs were replaced with the active ingredient names. For quantitative data, descriptive statistics, including means, standard deviations, and medians, with interquartile ranges (IQR), were calculated using Microsoft Office Professional Plus, Excel, version 16.0 (2016). For qualitative data, interviews were analyzed according to the grounded theory approach [17]. Thematic data saturation was reached after 24 interviews. The qualitative data analysis consisted of the following three processes: open coding, axial coding to define categories, and selective coding to identify core categories. MAXQDA Plus 2022 software (VERBI GmbH, Berlin, Germany, version 22.1.0) was used for open coding. Microsoft Office Professional Plus, Excel, version 16.0 (2016) was used for axial and selective coding. A.B. and M.S.F. independently coded the qualitative data within the four key themes. The core categories and the selected patient quotes were determined through discussion until consensus was reached.

### 2.5. Data Reporting

We reported our findings following the checklist for Good Reporting of a Mixed Method Study (GRAMMS) by O’Cathain et al. (2008) [18].

### 2.6. Quality Assessment of the Study Design

The methodological quality of the study was assessed using the Mixed Methods Appraisal Tool (MMAT, version 2018). The qualitative and quantitative components were each evaluated according to the MMAT mixed-methods criteria [19].

## 3. Results

### 3.1. Demographics

In 2023, 24 patients who had previously undergone a PGx test as part of a case series study in the past and therefore had access to PGx information were enrolled in the study. The median time between the PGx test and the study enrollment was 1 year [IQR 0–2]. The population was mainly female (71%), with a median age of 61 years [IQR 50–65]. The initial reason for PGx testing was primarily reactive due to DRPs in 20 patients (83%), while four patients (17%) underwent preemptive testing due to a positive family history, without experiencing DRPs themselves.

Among the reactive PGx tests, eight patients (40%) suspected drugs from the Anatomical Therapeutic Chemical (ATC) group N02A (opioids) as potential perpetrators of their DRPs, six patients (30%) suspected drugs from N06A (antidepressants), and four patients (20%) suspected drugs from B01A (antithrombotic agents). The main diagnoses were mental and behavioral disorders in nine patients (38%), musculoskeletal system and connective tissue diseases in seven patients (29%), and circulatory diseases in five patients (20%). Further details on patients’ characteristics are provided in Table S1.

The median time between the PGx test and the follow-up was 2 years [IQR 1–3]. The follow-up was completed between March and October 2024, primarily through telephone interviews with 20 patients (83%) with a median duration of 15 min [IQR 10–20]. The remaining four patients (17%) completed the semi-structured questionnaire in written form.

### 3.2. Medication Changes

During the follow-up period of 1 year, 18 participants (75%) experienced a medication change. At enrollment, patients were taking a median of four [IQR three–six] prescribed drugs. By the time of the follow-up, the number of prescribed drugs per patient increased to a median of five [IQR three–seven]. Overall, a total of 62 medication changes were identified. The most frequent type was the initiation of a new drug, accounting for 30 medication changes (55%), followed by 19 discontinuations (31%). Thirty-six medication changes (58%) were initiated by medical specialists, followed by 24 medication changes (39%) initiated by general practitioners (GP). Patients’ PGx information was considered for 15 patients in 39 medication changes (63% of all medication changes), with a median of two [IQR 2–3] per patient. Additional details on medication changes are provided in Table 1.

#### 3.2.1. Reason for Medication Changes

The patient-reported reasons for medication changes were qualitatively assessed through an open-ended question. Based on the analysis, three core categories reflecting the main reasons for medication changes were identified, as follows:

- Drug-related problems (DRP)

Patients expressed DRPs as a reason for medication changes. Mostly, **ADRs** were identified as DRPs. Patient (P)11 noted: “*I experienced severe headaches, dizziness, and nausea while taking voriconazole, which led to its discontinuation.*” In this context, the relationship between discontinuing one medication due to ADRs and initiating another was also described. P2 shared: “*I switched from atorvastatin to a combination drug with ezetimibe, which I tolerate much better now.*” In addition, **TF** was reported as another DRP that led to a medication change. P6 explained: “*Pantoprazole never worked for me; this was also confirmed by the pharmacogenetic test, so I switched to a different gastric protection medication.*”

- Change in health status

Changes in health conditions were identified as another reason for medication changes. Patients described the **deterioration of their health conditions**, highlighting the need **to treat newly diagnosed diseases**. P23 shared “*I started hydroxychloroquine after being diagnosed with lupus erythematosus.*” Additionally, medication changes were initiated **to optimize a pharmacotherapy due to persistent or aggravated symptoms**. P18 described “*I felt very tired and weak. Blood tests showed elevated thyroid levels, so the dosage of levothyroxine had to be increased.*” Similarly, P17 described “*I needed more support during opioid withdrawal, so clonidine was then prescribed. The doctor explained that, according to the pharmacogenetic test, clonidine is safe and suitable for me.*” Other patients expressed **the improvement of a health condition** as a reason for medication changes. P25 explained: “*My last flare-up was at Christmas 2022, about 1.5 years ago. I have been completely pain-free for the past 9 months, so I stopped taking prednisone.*”

- Concerns about medications in general

Some patients expressed **personal concerns** as a reason for medication changes due to the rejection of pharmacological treatments. P12 stated: “*I no longer want to take chemical substances.*” Similarly, P13 remarked: “*After 10 years, I simply no longer wanted to take ticagrelor.*”

#### 3.2.2. Preferences for Further Medication Changes

After asking about the reason for medication changes in the past year, we assessed whether patients currently desire further changes to their medication. The majority, 20 patients (83%), indicated that they did not wish to make any additional changes. However, four patients (17%) expressed a desire for further changes. The primary reason for seeking further changes was current DRPs, as exemplified by P23 “*In the long-term, I wish that the hydroxychloroquine is replaced because of recurrent diarrhea*”*,* and P9 “*Hardly any medication I have ever tried has been effective.*”

### 3.3. Evaluation of Current Treatment

Patients were asked to evaluate their current treatment. Following the qualitative analysis of the open-ended question, the following reasons for either treatment satisfaction or dissatisfaction were identified:

- Reasons for treatment satisfaction

Patients expressed that the **absence of symptoms** and therefore the sense of **wellbeing** led to treatment satisfaction. P3 stated: “*I am currently satisfied and have no complaints.*” Similarly, P20 shared: “*I had no complaints throughout the whole last year.*” Also, an **effective and well-managed drug therapy** was noted as a reason for satisfaction. P21 remarked: “*I feel well and I am very satisfied; the ketamine infusions help me a lot.*” Moreover, **positive interpersonal treatment experiences** were highlighted in the context of treatment satisfaction; P13 commented: “*I am receiving excellent care from the head of orthopedic and trauma surgery*”.

- Reasons for treatment dissatisfaction

Patients reported **ADRs** as a reason for dissatisfaction with their current drug therapy. P5 noted: “*I am experiencing side effects from Adalimumab*.” Other patients mentioned **new or persistent symptoms**, such as postoperative pain, as a reason for dissatisfaction. In this context, P13 described: “*Since the surgery in 2023, I have developed chronic pain in my hip and spine. It has been a very difficult year*.”

### 3.4. Use of PGx Information

Beyond its use relating to medication changes, 13 patients (54%) indicated that their PGx information was used over the past 12 months. Based on patients’ responses to an open-ended question, two core categories were identified, representing the settings and individuals involved in the utilization of PGx information, as follows:

- Utilization in clinical practice by HCPs

Patients reported that healthcare professionals (HCPs) actively considered PGx information. P8 shared: “*Yes, my PGx data was used at the cardiologist’s office when we considered starting a statin therapy.*” Some HCPs demonstrated a high level of interest in the implications of the PGx data and took it seriously. P17 stated: “*The doctors reviewed my genetic profile and took it very seriously.*”

In addition, interprofessional discussions between treating physicians about the patient’s PGx data were mentioned, as P23 noted: “*My previous rheumatologist reviewed the entire genetic profile with my new rheumatologist and explained what she should pay attention to.*”

Situations where PGx information was actively used included hospital stays and surgical procedures. P7 described: “*Before a shoulder surgery, the anesthesiologist and the surgeon reviewed the pharmacogenetic report. After the surgery, the report was also considered on-ward [..]. The doctors and nurses showed interest.*” Similarly, P13 highlighted the consistent consideration of the PGx report by the anesthesiologists: “*Most didn’t pay much attention to the genetic profile, except for the anesthesiologists. They were the only ones who took it very seriously. Over my hospital stay, I had six surgeries, and every time, the anesthesiologists consulted the profile again.*”

Following anesthesiologists, surgeons, and cardiologists, endocrinologists, rheumatologists, and neurologists were also mentioned as HCPs who actively used the PGx information.

- Utilization and proactive incorporation by patients

Patients reported consulting their PGx data independently. P4 stated: “*I use the PGx report by myself; my doctor doesn’t.*” Other patients confirmed that PGx information was considered by HCPs but only at their proactive request. P9 expressed: “*It is only reviewed by the doctor when I specifically point it out, […] It is also archived at my pharmacy, but it is never actually considered unless I remind them*”. Similarly, P10 explained: “*The genetic profile wasn’t actively used. My new GP received it from my previous GP and stored it in their files. He only looked at it because I asked him to.*”

### 3.5. Non-Use of PGx Information

Based on the qualitative assessment of an open-ended question, four core categories of patient-reported factors contributing to the non-use of PGx information were defined as follows:

- Insufficient knowledge among HCPs

Patients reported that one reason for the non-use of PGx information is the **knowledge deficit** among physicians and pharmacists in this area. P3 remarked: “*I have the feeling that doctors don’t understand anything about medications*”*,* while P9 noted: “*There is a knowledge gap regarding the existence of pharmacogenetic information. Especially in pharmacies, I find that hard to understand.*”

Additionally, the **lack of awareness** and the **need for further education** were emphasized in the context of insufficient knowledge. P17 stated: “*Doctors need more information about pharmacogenetics, how to use it concretely in everyday practice, and what to do with the information.*” Similarly, P19 observed: “*Sometimes I feel like doctors are not even aware of it, so of course they do not understand it.*”

- Lack of interest among HCPs

Patients perceived **irrelevance**, **skepticism**, and **disinterest** from HCPs. P11 shared: “*My GP was skeptical. He was familiar with pharmacogenetics and knew its importance but said that nothing serious would happen if I kept taking voriconazole and that just more side effects would occur*.”

The **lack of interdisciplinary collaboration** and **reliance on isolated approaches** were also criticized. P6 stated: “*Many doctors do not even look at what they are sent. They immediately think it is irrelevant to their daily work and stick to what they are used to, just referring patients back and forth*.” Similarly, P15 explained: “*My GP never properly reviewed the information. Pharmacogenetics is research, and if doctors aren’t open to research, they don’t realize how central it is*.” Patients further noted that initial interest in PGx information diminished over time. P24 reflected: “*When the test results were new, the doctors found them interesting, but after some time, no one ever brought it up again*.”

- Structural and administrative barriers

Patients described that physicians are reserved in adapting individualized processes and that they **rely on established workflows**. P20 commented: “*It’s too individual for a GP.*” Similarly, P20 noted: “*Unfortunately, GPs tend to rely on the standard therapy.*” Others criticized outdated practices, as P7 mentioned: “*They follow the old school thoughts, they are set in their ways.*” Patients also felt that PGx information was not used in the first place to inform therapy decisions but rather as the last option. P19 shared: “*I think doctors always try what has worked best for most of their patients. They focus less on the individual patient and stick to what they know. If that doesn’t work, then they may consider other options such as the pharmacogenetic report.*”

Moreover, many patients argued that the consideration of PGx information is **time-consuming**. P13 remarked: “*It’s a lot of work to engage with this, and doctors simply don’t have the time.*” Similarly, P15 observed: “*GPs just don’t have time for this.*” P16 elaborated: “*It requires effort to familiarize with the subject. I think doctors feel that the standard approach has worked well so far. So why change? Individualization always requires effort, and anything time-consuming is hard to implement.*”

In addition, patients negatively emphasized the structural barrier of having to rely on their own **initiative and responsibility**. P9 explained: “*If you remind them that it is important to you, they usually take note, but they don’t check on their own. This is especially problematic for those who are unable to advocate for themselves*”.

- Situational absence of need

Some patients reported no need to use the PGx information as they were satisfied with their current treatment and had no symptomatic complaints. P10 explained: “*It’s not that the genetic profile was intentionally disregarded, there was simply no situation where it was needed over the past year. My medications are well-adjusted, and I have no complaints.*” Similarly, P16 remarked: “*In my case, there was no need to apply the genetic information last year. Everything was and is going well.*”

## 4. Discussion

### 4.1. Long-Term Use of PGx Information to Inform Treatment Decisions

The study results demonstrate that PGx information was considered for medication changes, with a median of 2 years between PGx test and follow-up. This suggests that PGx information is not only used when addressing the initial reason for testing, but it is also reused over time, highlighting its potential for long-term utility. In a previous analysis of a Swiss case series study, we observed that PGx information was considered in 62% of all medication changes within 6 months after a PGx test [15]. Comparably, PGx information was considered in 63% of all medication changes during this longer follow-up period, suggesting that the consideration of PGx information to inform treatment decisions does not diminish over time. However, in this patient population with main diagnoses in the International Classification of Diseases, 10th Revision (ICD-10) sections F, M, and I, a high compliance rate in using PGx information might be expected based on findings from the literature [20,21,22,23]. Various drugs used to treat these diagnoses are PGx-actionable, which means that concrete dosing and recommendation guidelines exist (www.pharmgkb.org, accessed on 30 January 2025). Consequently, the implications of PGx testing results may be more relevant for physicians of these patient groups.

In our study, a median of four medication changes per patient were initiated between enrollment and follow-up, with a median of two medication changes involving the consideration of PGx information, as perceived by the treated patient. This represents a relevant use of PGx information, especially important in this patient population at risk for polypharmacy. The high percentage of the initiation of a new drug as the main type of medication change, along with the increasing median number of prescribed drugs per patient between enrollment and follow-up, demonstrates that the expected growing number of daily medication use is already evident in a patient population with a median of 61 years.

Beyond the explicit PGx-based recommendation to change a medication, patients reported that PGx information was also considered, even when those decisions to change a medication were initiated for other reasons, such as changes in health status. This suggests that having access to PGx panel test information is helpful regardless of the context, even to validate that a drug is pharmacogenetically not relevant (e.g., as P17 noted during opioid withdrawal and the initiation of clonidine).

However, it is essential to note that PGx does not address all DRPs, and symptoms may persist even when PGx information is considered to inform treatment decisions. In this context, four patients of the study population expressed a desire for further medication changes, as they were experiencing an ADR or TF from medications that are not known to be PGx relevant (e.g., P23 with hydroxychloroquine). Drug response is influenced by numerous factors, many of which cannot be explained by PGx [24,25].

Of the study population, 17% expressed a desire for further medication changes due to dissatisfaction with their current drug therapy caused by DRPs, while 83% reported being satisfied. Notably, Kemp et al. (2020) described in a systematic review that 10–20% of hospital admissions among older patients (>65 years) are attributable to DRPs [26]. Given the demographic similarity, the observed 17% of patients experiencing DRPs in our study may be representative of this patient group. This is in line with a study by Reji et al. (2024) of older patients, reporting that 75% were satisfied with their overall medical care, while 25% were dissatisfied [27]. These findings also broadly align with our study results. Reji et al. (2024) further found that patients with higher literacy status were more satisfied, which could be attributable to greater awareness of treatment options and their benefits. Since all our patients underwent PGx testing and counseling, it could be hypothesized that they are, in general, well-informed about their medications and PGx data. This might reflect a higher health literacy level, which, according to the literature, is associated with better health outcomes [28,29].

### 4.2. Patient-Reported Factors Enabling the Utilization of PGx Information

The results provided valuable insights into the use of PGx information and evaluated patient-reported factors for its non-use. These findings serve as a basis for identifying enablers for the long-term implementation of PGx in clinical practice.

#### 4.2.1. Need for Interprofessional Collaboration, Education, and Awareness

An important finding is the need for improved interprofessional collaboration among HCPs. Our study participants highlighted no or limited communication between HCPs and the inconsistent sharing of PGx data across specialties, as structural and systemic barriers. These observations have previously been reported in the literature [30,31]. Also, Waldman et al. (2019) emphasized that the inconsistent integration of PGx data into care processes across different specialties represent a key limitation in the effective application of PGx information [32]. This suggests that effective interprofessional collaboration, particularly between GPs, medical specialists, and pharmacists, might be essential to maximize the utility of PGx data in clinical workflows.

The lack of awareness and education among HCPs was recognized by our study participants as a reason for the non-use of PGx data. These findings indicate a need for expanded training opportunities for both undergraduate and postgraduate HCPs. This aligns with prior research, which has demonstrated that targeted educational initiatives improve HCPs’ confidence and competence in handling PGx information [33,34]. Adesta et al. (2021) showed that pharmacogenomics implementation training enhances HCPs’ self-efficacy, which may facilitate its adoption in clinical workflows [33]. Similarly, Stäuble et al. (2021) reported the positive impact of a digital blended learning program on pharmacist-led PGx services [34].

While the knowledge gap among HCPs is only a patient-reported perspective in our study, the existing literature addresses physicians’ and pharmacists’ attitudes toward PGx, revealing a self-reported lack of awareness and education [35,36]. Muflih et al. (2021) explored in a cross-sectional survey of 200 physicians their self-reported knowledge gaps in PGx. The majority of the physicians expressed that they were not aware of PGx testing and uncertain about their expertise in implementing PGx testing results [35]. In addition, Makrygianni et al. (2023) investigated pharmacy students’ attitudes and intentions towards PGx, reporting that while interest was high, students felt unprepared to use PGx in clinical practice due to insufficient training [36]. These findings suggest that the use of PGx data depends not only on the information itself but also on the educated and supportive environment in which it is applied. This highlights the need to integrate PGx education on a broader scale into healthcare systems, which is also supported in the literature by calls for systemic reforms to promote awareness among all stakeholders, including policymakers [33].

#### 4.2.2. Patient Empowerment Without Shifting Responsibility

Participants in our study demonstrated a proactive initiative to ensure their PGx information was considered in treatments decisions. This observation reflects a study finding that highlights the importance of patient empowerment as an essential cornerstone of modern medicine [37]. The proactive involvement of patients who independently consulted their PGx data or asked HCPs to consider it demonstrates how patients’ engagement can positively influence PGx information utilization. However, our results indicated that not all patients want to take an active role in coordinating their care (e.g., P10, P13). In the literature, the over-reliance on patients’ self-advocacy has also raised concerns [38,39,40]. Ramos Salazar (2018) emphasizes that not all patients feel comfortable or capable of advocating for themselves, particularly those facing barriers (e.g., severe illness, immobility, or communication deficits) [40].

The dual role of patient empowerment as both an enabler and a potential barrier to PGx utilization highlights the need for systems to ensure that PGx data are seamlessly integrated into clinical care and proactively used by HCPs without requiring patients to advocate for themselves. This might be particularly important for patients who are unable to do so due to severe illness, language, or other barriers. However, since our study revealed that some patients use the PGx information independently (e.g., P4), PGx testing providers should offer educational materials and resources to ensure patients understand the role and limitations of PGx information accurately.

#### 4.2.3. Practical Utility of PGx Information

The utility of practical PGx recommendations emerged as a key enabler in informing treatment decisions. This was notably shown in scenarios where the data directly informed treatment decisions, as described by patients who reported active consideration by HCPs (e.g., P8). Another driver was the interest and engagement shown by HCPs when reviewing the PGx information. Patients mentioned that certain medical specialists, particularly anesthesiologists, incorporated PGx information into their decision-making during hospital stays and surgical procedures. This suggests that integrating PGx into specific clinical workflows, such as perioperative care, could serve as a model for broader adoption. The utilization of PGx information by anesthesiologists in PGx data has previously been observed in the literature [41,42,43]. Gabriel et al. (2020) and Landau et al. (2012) identified the perioperative care as a promising area for PGx integration [42,43]. These findings align with literature emphasizing the utility of PGx data in contexts where clinical relevance has already been described. Crews et al. (2021), Matic et al. (2022), and Hicks et al. (2017) elaborated on how dosing guidelines for PGx-actionable drugs, specifically analgesics, opioids, and antidepressants, facilitate their direct application in clinical practice [44,45,46]. This perspective is supported by the existing literature, which suggests that excessive or ambiguous information may hinder the clinical implementation of PGx [33,46]. These findings indicate that the utilization of PGx information and, therefore, the success of long-term PGx implementation also relies on how well the information is presented and tailored to relevant clinical questions by the provider. We suggest that PGx information should be structured, concise, and focused on PGx-actionable drugs with established guidelines or at least robust evidence, while prioritizing clarity and relevance over excess information.

### 4.3. Study Limitations and Future Directions

This study has limitations that should be considered. First, all findings are based on patients’ perspectives, which provide valuable insights, but may lack the complementary point of view of other involved parties, especially of HCPs. However, we already conducted a study investigating the HCPs’ perspective on the implementation of PGx [47]. The findings of our study and those of Wiss et al. (2024) revealed both shared and diverging perspectives regarding the barriers and enablers in the implementation of PGx in clinical practice. One key similarity is the recognition of insufficient PGx knowledge among HCPs. Also, both studies highlighted the importance of interprofessional collaboration as a critical enabler, while its absence was identified in both studies as a barrier. A notable divergence between the two studies concerns the role of patient empowerment. Wiss et al. (2024) described that HCPs desired greater patient involvement as a facilitator for successful PGx implementation. In contrast, our study reported that patients often perceived this as a burden, particularly when they were required to advocate for the use of PGx information. However, it is important to note that the two studies are not directly comparable, as their methodologies and the questions posed to participants differed. Future directions could include the establishment of a focus group comprising all involved stakeholders, including physicians, pharmacists, and patient representatives, to foster a comprehensive dialogue and share the diverse perspectives and needs of HCPs and patients regarding the implementation of PGx.

Second, the sample size consisted of 24 participants, and, while thematic saturation was reached, the selective patient recruitment may have limited the generalizability of our findings. In this context, it must be noted that sociodemographic factors were not collected. As a result, it is possible that the educational attainment of our study population does not represent that of the general population [48], particularly since our findings indicate that our population exhibits a high level of health literacy. Also, it may be possible that our study participants were primed to use their PGx testing results more frequently due to the enrollment in the study.

Third, as the study inclusion criteria did not define restriction on the time frame since the PGx test, the test was conducted at various timepoints among the patients. Conclusions about the longitudinal use of PGx information following testing were derived from the median values. Further, the follow-up period was limited to 1 year after study enrollment. To ensure transferability of our results, follow-ups should be continued for 3–5 years after PGx testing to compare whether the use of PGx information in informing treatment decisions decreases or stabilizes over time. Moreover, the insights gained from this study will serve as a basis for further development of a structured questionnaire, enabling an enhanced continuation of this research. Beyond this, the next steps should include investigating the economic and clinical outcomes of PGx-guided therapy to further strengthen the motivation for integrating PGx into clinical practice.

## 5. Conclusions

Our findings demonstrate that PGx information is not only used immediately after initial testing but is also continuously used to inform treatment decisions over time. This highlights its potential for sustained clinical relevance. Based on the patient-reported factors for the use or non-use, we identified several potential enablers for the successful and long-term integration of PGx information into routine clinical workflows, including the need for interprofessional collaboration among HCPs, enhanced PGx education, and raising general awareness about personalized pharmacotherapy, particularly among policymakers. Simultaneously, while patient empowerment is becoming increasingly important for modern medicine, technical solutions could ensure that patients’ PGx information is used by HCPs without relying on patients’ self-advocacy. To improve the practical utility of PGx information, testing providers should ensure that recommendations are well-structured, concise, and focused on PGx-actionable drugs with established evidence. By addressing these interdependent challenges, PGx can evolve into a clinically integrated cornerstone of individualized pharmacotherapy, ultimately improving patient care over the long term.

## Figures and Tables

**Table 1:** Overview of patients’ medication.

Characteristic	Category	Value
Number of prescribed drugs, median [IQR], maximum, minimum	Enrollment Follow-up	4 [3–6] 13, 1 5 [3–7] 14, 0
Medication changes between enrollment and follow-up, *n* Median [IQR] Maximum, minimum	Total Per patient	62 4 [2–4] 8, 1
PGx-considered medication changes ^1^ between enrollment and follow-up, *n* Median [IQR] Maximum, minimum	Total Per patient	39 2 [2–3] 6, 1
Type of medication change, *n* (%)	Initiation of a new drug; Discontinuation of a drug; Start/Stop of a new drug ^2^; Dosage increase.	34 (55%) 19 (31%) 4 (6%) 5 (8%)
Initiation of medication change, *n* (%)	Medical specialist; General practitioner; Patient.	24 (58%) 36 (39%) 3 (3%)

^1^ PGx-considered medication change: The consideration of PGx information for medication changes is defined as the consultation of the PGx information prior to a medication change to determine whether a clear warning exists to initiate the change or to confirm the absence of any warnings. ^2^ Start/Stop of a new drug: Initiation of a new drug, which was discontinued after six months. Abbreviation: IQR, interquartile range; n, number; PGx, pharmacogenetics.

## Data Availability

The data presented in this study are available on request from the corresponding author.

## Supplementary Materials

The following supporting information can be downloaded at: https://www.mdpi.com/article/10.3390/pharmacy13010014/s1, Table S1: Patients’ characteristics. Supplementary Materials File S1: Semi-structured interview guide.

## Abbreviations

The following abbreviations are used in this manuscript:

ADRs	Adverse drug reactions
ATC	Anatomical therapeutic chemical
DRPs	Drug-related problems
GP	General practitioner
GRAMMS	Good Reporting of a Mixed Method Study
HCP	Healthcare professional
ICD-10	International classification of diseases, 10th revision
IQR	Interquartile range
MeSH	Medical Subject Headings
MMAT	Mixed Methods Appraisal Tool
MRT	Magnetic resonance imaging
PD	Pharmacodynamics
PGx	Pharmacogenetics
PK	Pharmacokinetics
TF	Therapy failure
UK	United Kingdom
