# Supplementary material for: Impact and Enablers of Pharmacogenetic-Informed Treatment Decisions—A Longitudinal Mixed-Methods Study Exploring the Patient Perspective
Source: Pharmacy (Basel). 2025 Jan 31;13(1):14. doi: 10.3390/pharmacy13010014 (PMC11859433; doi:10.3390/pharmacy13010014)
Supplement: Supplementary file 1 [file pharmacy-13-00014-s001.zip › Supplement S1.pdf]

Supplement S1

Table S1. Patients' characteristics.

**Characteristic**	**Category**	**Value**
Subjects, n		24
Age (years), median [IQR],<br>maximum, minimum		61 [50-65]<br>78, 29
Gender, n (%)	Female<br>Male	17 (71%)<br>7 (29%)
Main diagnosis, n (%)	Mental and behavioral disorders (ICD-10: F)<br>Diseases of the musculoskeletal system and connective tissue (ICD-10: M)<br>Diseases of the circulatory system (ICD-10: I)<br>Others[1]	9 (38%)<br>7 (29%)<br>5 (20%)<br>3 (13%)
Initial reason for PGx testing, n (%)	Reactive<br>Preemptive	20 (83%)<br>4 (17%)
Suspected ATC-group for reactive testing, n (%)	B01A: Antithrombotic agents<br>C07A: Beta blocking agents<br>C10A: Lipid modifying agents<br>M01A: Anti-inflammatory and antirheumatic products, non-steroids<br>N02A: Opioids<br>N06A: Antidepressants	4/20 (20%)<br>2/20 (10%)<br>1/20 (5%)<br>3/20 (15%)<br>8/20 (40%)<br>6/20 (30%)
Time since PGx testing (years), median [IQR]<br>maximum, minimum		2 [1-3]<br>5, 1
Time spend for follow up in minutes, median [IQR]<br>maximum, minimum		15 [10-20]<br>30, 10

[1]Other diseases include diagnoses of ICD-10: E (endocrine, nutritional and metabolic diseases), and ICD-10: H (diseases of the eye and adnexa).

Abbreviation: ATC, anatomical therapeutical chemical; ICD-10, International Classification of Diseases 10th Revision; IQR, interquartile range; n, number.
