# Supplementary material for: Impact and Enablers of Pharmacogenetic-Informed Treatment Decisions—A Longitudinal Mixed-Methods Study Exploring the Patient Perspective
Source: Pharmacy (Basel). 2025 Jan 31;13(1):14. doi: 10.3390/pharmacy13010014 (PMC11859433; doi:10.3390/pharmacy13010014)
Supplement: Supplementary file 1 [file pharmacy-13-00014-s001.zip › Supplement S2.pdf]

Supplement S2: Semi-structured interview guide

Introduction

Hello, my name is _________________.

I am a __________ at the University of Basel and I am contacting you regarding your participation in the study "Pharmacogenetic Information for Medication Optimization Over Time." We are now conducting the first follow-up interview as part of the study.
(Pause for greeting)

We will, of course, treat your information confidentially. You may pause, stop, or refuse to answer any question at any time during the interview. If any of the questions are unclear, please feel free to ask. I will be happy to answer any additional questions at the end of the interview.

Shall we begin? (...)

1. Has the medication plan been changed in the past 12 months? Yes ☐ No ☐

(If yes, complete the table for each medication change – If no, proceed to question 2)

Date of change	Medication	Dosage	Type of change* (Nr. 1-6)	Initiated by** (GP, MS, PH, P)	Reason for change***	Consideration of PGx information?
						Yes ☐ No ☐

*The type of change is assessed structurally as defined according to the following numbers:
Nr. 1 - Initiation of a new medication
Nr. 2 - Discontinuation of a new medication
Nr. 3 - Start/Stop: Initiation of a new medication, but discontinuation of the same medication within the following 12 months
Nr. 4 - Increase in dosage
Nr. 5 - Reduction in dosage
Nr. 6 - Others

**GP = General Physician; MS = Medical Specialist; PH = Pharmacist, P = Patient

***The reason for change is assessed qualitatively based on the patient's response.

2. Do you desire (further) changes to your medication? Yes ☐ No ☐
- ➢ If yes, why?

3. Are you currently satisfied with your medication?

4. Has the information on your genetic profile been used independently of medication changes in the past 12 months? Yes ☐ No ☐
- ➢ If yes, in which context and by whom?

5. Could you think of reasons why your genetic information has not been routinely used in clinical practice?
